# Unraveling the interplay of circadian rhythm and sleep deprivation on mood: A Real-World Study on first-year physicians

**DOI:** 10.1371/journal.pdig.0000439

**Published:** 2024-01-31

**Authors:** Benjamin Shapiro, Yu Fang, Srijan Sen, Daniel Forger

**Affiliations:** 1 Department of Psychiatry, Dartmouth Health, Hanover, New Hampshire, United States of America; 2 Dartmouth College Geisel School of Medicine, Hanover, New Hampshire, United States of America; 3 Michigan Neuroscience Institute, University of Michigan, Ann Arbor, Michigan, United States of America; 4 Department of Psychiatry, University of Michigan, Ann Arbor, Michigan, United States of America; 5 Department of Mathematics, University of Michigan, Ann Arbor, Michigan, United States of America; 6 Department of Computational Medicine and Bioinformatics, University of Michigan, Ann Arbor, Michigan, United States of America; University of Cagliari: Universita degli Studi Di Cagliari, ITALY

## Abstract

The interplay between circadian rhythms, time awake, and mood remains poorly understood in the real-world. Individuals in high-stress occupations with irregular schedules or nighttime shifts are particularly vulnerable to depression and other mood disorders. Advances in wearable technology have provided the opportunity to study these interactions outside of a controlled laboratory environment. Here, we examine the effects of circadian rhythms and time awake on mood in first-year physicians using wearables. Continuous heart rate, step count, sleep data, and daily mood scores were collected from 2,602 medical interns across 168,311 days of Fitbit data. Circadian time and time awake were extracted from minute-by-minute wearable heart rate and motion measurements. Linear mixed modeling determined the relationship between mood, circadian rhythm, and time awake. In this cohort, mood was modulated by circadian timekeeping (p<0.001). Furthermore, we show that increasing time awake both deteriorates mood (p<0.001) and amplifies mood’s circadian rhythm nonlinearly. These findings demonstrate the contributions of both circadian rhythms and sleep deprivation to underlying mood and show how these factors can be studied in real-world settings using Fitbits. They underscore the promising opportunity to harness wearables in deploying chronotherapies for psychiatric illness.

## Author summary

Our body has an internal clock that controls much of our physiology. New smartwatch technology allows researchers to study this clock without limiting subjects to a specific environment. In this study, we examine how daily mood in first-year doctors changes based on their internal clock and sleep/wake cycle. Over a two-year period, we collected daily mood survey scores and Fitbit data from more than 2,500 first-year doctors. We discovered that mood follows a rhythm connected to this clock, and the clock’s influence on mood increases as someone stays awake longer. This study highlights the significant role our body’s clock plays in mood and introduces wearable technology as an exciting new way to explore these factors in mental health issues.

## Introduction

Internal 24-hour (circadian) biological clocks regulate many physiological processes in an organism such as metabolism, hormone secretion, and the sleep-wake cycle. Circadian rhythms are any physiological process that follows a 24-hour cycle and are primarily regulated by these biological, or circadian, clocks. Clock genes have been found to be expressed in cells throughout the body and these cells express an internal rhythm that in turn induce regulatory circadian rhythms on local tissue and organs [1–3]. The central clock within the human body resides in the suprachiasmatic nucleus of the brain and acts to synchronize all these peripheral clocks [4].

Circadian rhythms are distinct from sleep-wake cycles or sleep deprivation because of their primary regulation through biological clocks rather than environmental cues. These rhythms endure even in the absence of external cues or environmental influences as they are inherently driven by internal mechanisms. The difference is illustrated with the flexibility of sleep/wake schedules in night shift workers. In these individuals, circadian clocks measured through endogenous temperature and plasma melatonin levels do not always re-align to match the shift work schedule [5, 6].

Circadian rhythms, sleep-wake cycles, and sleep deprivation all affect physiology. Misalignment of these rhythms can lead to adverse health outcomes such as psychiatric and metabolic disorders, cardiovascular disease, and cancer [7, 8].

One important aspect of our physiology which is under the control of circadian timekeeping is mood. Mood is the constantly changing emotional lens which we see and interact with our environment. Adverse mood can lead to many health challenges including cardiovascular disease, diabetes, and suicide [9–11]. Thus, a careful understanding of mood is important for optimal health.

There has been much interest in connecting mood, circadian rhythms, and time awake in healthy individuals. It has been shown that there is a diurnal variation in mood and that mood deteriorates with sleep deprivation [12–15]. Furthermore, it has been demonstrated that mood varies with circadian phase and that, depending on the phase, mood either improved or worsened based on time awake [16]. However, such studies were in controlled conditions, had smaller number of subjects, or may not have used recent statistical methods [12, 17–20].

Additional evidence highlighting the close relationship between mood and the circadian pacemaker can be observed in individuals experiencing mental health conditions marked by mood disturbances. In such cases, circadian rhythms have been found to be disrupted, with the type of this impact differing based on the specific disorder. In the case of depression, cortisol and temperature measurements have indicated a phase delay accompanied by a reduction in rhythmic amplitude [21, 22]. Mood displays an alternative diurnal variation in these individuals. Instead of peaking in the late afternoon as seen in healthy individuals, it progressively rises throughout the evening [15, 23]. Mania, which is marked by a significant decrease in sleep requirement along with an abnormally heightened mood, has been linked to a phase advance in the biological clock [24].

Recently, genetic risk factors for affective disorders have been identified. The a-kinase anchoring protein 11 (AKAP11) gene emerged as a risk factor for bipolar disorder with this variant occurring 7 times higher in this population [25]. AKAP11 interacts with glycogen synthetic kinase 3 (GSK3B) which is a key component of the circadian clock that modulates neuronal excitability through the regulation of persistent sodium channels [26].

Studying circadian rhythms in humans has previously required invasive laboratory monitoring such as minute by minute rectal temperature measurements or frequent blood draws [27]. These measurements can require participants to be confined to a laboratory setting for days or even weeks. While some of the methods have been adapted to non-laboratory settings, they still require subjects to spend hours in darkness and costly and time-consuming biochemical assays. Such methods can remove many social factors that could affect mood and circadian timekeeping. However, it is unclear how being removed from society could affect mood. Additionally, such biochemical assays cannot scale to understand population levels interactions between sleep, circadian rhythms and mood.

In the real-world, individuals encounter a myriad of daily social challenges that are absent in laboratory settings. Due to the numerous confounding factors, it remains uncertain whether a significant relationship between circadian rhythm, time awake, and mood can be established in everyday life.

Investigations into peripheral rhythms have shown a circadian rhythm of heart rate and heart rate variability [28–33]. Data collection in these studies required either a sleep laboratory or portable electrocardiograms placed with clinical supervision. Advances in wearable technology have made continuous heart rate, sleep stage, and activity monitoring possible for the general population. Algorithms created by Bowman et al. have further allowed extraction of circadian phase from wearable data without the need for invasive temperature or laboratory monitoring [34]. The ability to measure circadian rhythm noninvasively has provided an opportunity to investigate the relationship between the biological clock and mood amidst the unpredictable and tumultuous nature of daily life.

Here, we study the relationship between circadian timekeeping, sleep and mood using this new mobile and wearable technology. A key question is whether historical laboratory findings in clinical settings would generalize to real-world settings. This technology could help in the treatment of mood; if mood is unduly lowered by sleep deprivation or circadian timekeeping, simple circadian or sleep interventions would be appropriate. It could also indicate abnormal mood patterns, even when sleep and circadian rhythms are accounted for, to inform psychiatric diagnosis, suicide prediction, and treatment decisions.

## Methods

### Participants

The Intern Health Study is a multicenter study across the United States involving first year physicians [35]. Physician interns were recruited for this study through emails that were sent to both their medical school and upcoming place of internship. Recruitment lasted from April through June prior to the start of their internship. Participants were required to have an iPhone and were incentivized by receiving a Fitbit Charge 2 device and up to US $125, split into 5 distributions for continued participation [36]. Interns recruited in years 2018 and 2019 were included in this study.

Participants downloaded a mobile application which would prompt them with daily ecological momentary assessments [37]. Users were sent a daily push notification at a user-specified time between 5pm and 10pm reminding them to complete the once daily assessment. The assessment contained one question asking the user ‘on a scale of 1–10 how was your mood today?’ with a slider to select the score. While users received a push notification at a designated time, they had the ability to complete the survey at any point in the day. S1 Fig displays the mood scale. In addition, minute by minute sleep, heart rate, and step counts were collected while they wore the provided Fitbit device [38, 39].

A total of 489,523 surveys from 4,919 participants were collected. Of those, survey scores that did not contain at least 24 hours of abutting wearable data were removed. Participants were included in the study if they had at least one survey score with wearable data. A total of 168,311 survey scores met these criteria across 2,602 participants. Demographic data of age, sex, and ethnicity were collected by self-reporting on enrollment and displayed in Table 1 for these participants.

**Table 1:** Demographic breakdown of sample. The number of participants analyzed in this study was 2,602. Baseline characteristic proportions are rounded to nearest percentage.

Baseline Characteristic		Sample Proportion
Sex		
	Female	55%
	Male	45%
Ethnicity		
	Caucasian	61%
	Asian	21%
	African American	5%
	Latino	4%
	Mixed	9%
Age		
	Under 25	2%
	25 to 29	80%
	30 to 34	15%
	35 and above	3%

This raw data was automatically uploaded from participant Fitbit devices to a secure server at regular intervals during collection. The data was analyzed post-hoc on these servers after collection was completed using MATLAB [35] and Python [40] scripts. This study was approved by the University of Michigan IRB and all subjects were provided informed consent after receiving a complete description of the study.

### Statistical analysis

Bowman et. al. developed a model of heart rate informed by a comprehensive literature review that depicts heart rate as the combination of influences from physical activity, a 24-hour rhythm, and a standardized model of error [34].


$${HR}_{t}=a-b∙\cos\left(\frac{\pi }{12}(t-c)\right)+d\cdot activity+\varepsilon_{t}$$

The model of heart rate at time t (*HR*_*t*_) is governed by six physiological parameters–basal heart rate (*a*), amplitude of a 24-hour circadian oscillation in heart rate (*b*), time of the circadian heart rate minimum (*c*), increase in heart rate per step (*d*), and an autoregressive error function (*ε*_*t*_). The error function accounts for both the inaccuracies of the wearable device [41] and external factors such as cortisol and other hormones [42] which are driven by stimuli such as light, sleep cycles, stress levels, and meals [43–47].

To utilize Bowman et. al.’s model, heart rate and step data were averaged into 5-minute bins and data was separated by periods of sleep more than 2 hours in length. The model was then fit using Goodman and Weare affine invariant Markov chain Monte Carlo algorithm [48] to two wake-sleep intervals at a time. Each mood survey score was subsequently paired with an individual’s circadian heart rate minimum during the day of survey completion and their duration of prior time awake.

Circadian phase was then computed as the angle between the heart rate minimum and the time of survey completion. Circadian phase is the current state of the body’s internal clock. It is measured from 0° to 360° with 0° representing the heart rate minimum. On someone habituated to normal day-night conditions (entrained) this minimum occurs at the midpoint of sleep. A total of 168,311 survey scores were paired from 2,602 participants. For each score, a standardized mood z score was calculated.

To assess if mood changes based on circadian phase and time awake, linear mixed modeling was applied. Linear mixed modeling was chosen given the unbalanced study design and the serial mood measurements. Subjects were considered a random effect with fixed slope and random intercept. Given the circular nature of circadian phase, e.g. 0° = 360°, phase was modeled as the additive process of a sine and cosine wave. For each subject, mood was then modeled as the sum of the fixed effects of circadian phase, time awake, age, sex, and ethnicity.

$${Mood}_{ij}=\gamma_{00}+\gamma_{10}\;\cos\left({phase}_{ij}\right)+\gamma_{20}\;\sin\left({phase}_{ij}\right)+\gamma_{30}({cumulative\;time\;awake}_{ij})+\gamma_{40}({age}_{j})+\gamma_{50}({sex}_{j})+\gamma_{60}({ethnicity}_{j})+u_{0j}+e_{ij}$$

Where *i* represents each survey nested to subject *j*. *γ* is the coefficient for each fixed effect of the model with *u* denoting the random component of the intercept.

Using R packages lme4 and lmerTest [49, 50], coefficients and their p-values were calculated. The model was compared to one without the fixed effects of age, sex, and ethnicity. Using the R anova function, the fit of the model including demographic data was compared against one without.

To help understand the nonlinear relationship of time awake and circadian phase on mood, survey scores were broken into 4 strata. Strata were set at less than 6 hours, 6 to 12 hours, 12 to 18 hours, and 18 to 24 hours of time awake. Each stratum was then fit with its own model, however, now without the fixed effect of time awake.


$${Mood}_{ij}=\gamma_{00}+\gamma_{10}\;\cos\left({phase}_{ij}\right)+\gamma_{20}\;\sin\left({phase}_{ij}\right)+u_{0j}+e_{ij}$$

where *i* represents each survey nested to subject *j*. *γ* is the coefficient for each fixed effect of the model with *u* denoting the random component of the intercept.

The coefficients and the associated p-values were calculated in each stratum. Using only the fixed effect components of each model, we calculated the corresponding function amplitudes, minimums, and intercepts.

### Results

We analyzed a total of 168,311 survey scores from 2,602 participants. The sample was 55% female and 60% Caucasian. The majority of the subjects, 80%, were between the ages of 25 and 29. A comprehensive demographic breakdown is provided in Table 1 for further illustration.

**Fig 1:**
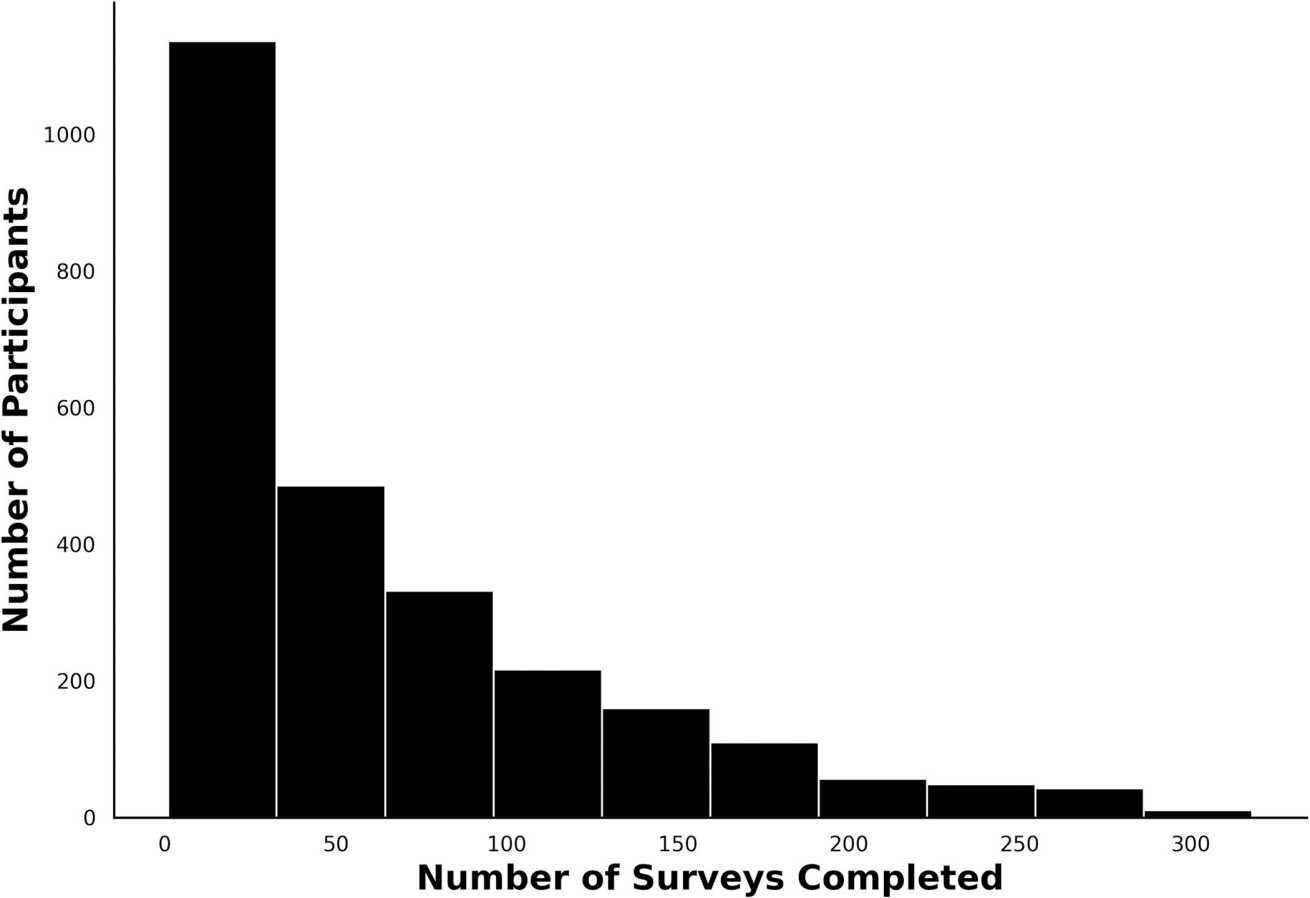
Distribution of Surveys Completed per Participant, showcasing the number of surveys each participant completed that were used in the statistical analysis of this study.

Each subject completed an average of 64.7 surveys. A histogram of the number of surveys per participant is displayed in Fig 1. The mean of the median time difference between successive surveys per participant was 2.58 days with a standard deviation of 2.41 days and mode of 1 day. Raw mood survey scores ranged from 1 to 10 with an overall mean of 7.3 and standard deviation of 1.5. Individual average mood scores spanned from 1.7 to 10 with standard deviation ranging from 0.07 to 3.09.

**Fig 2:**
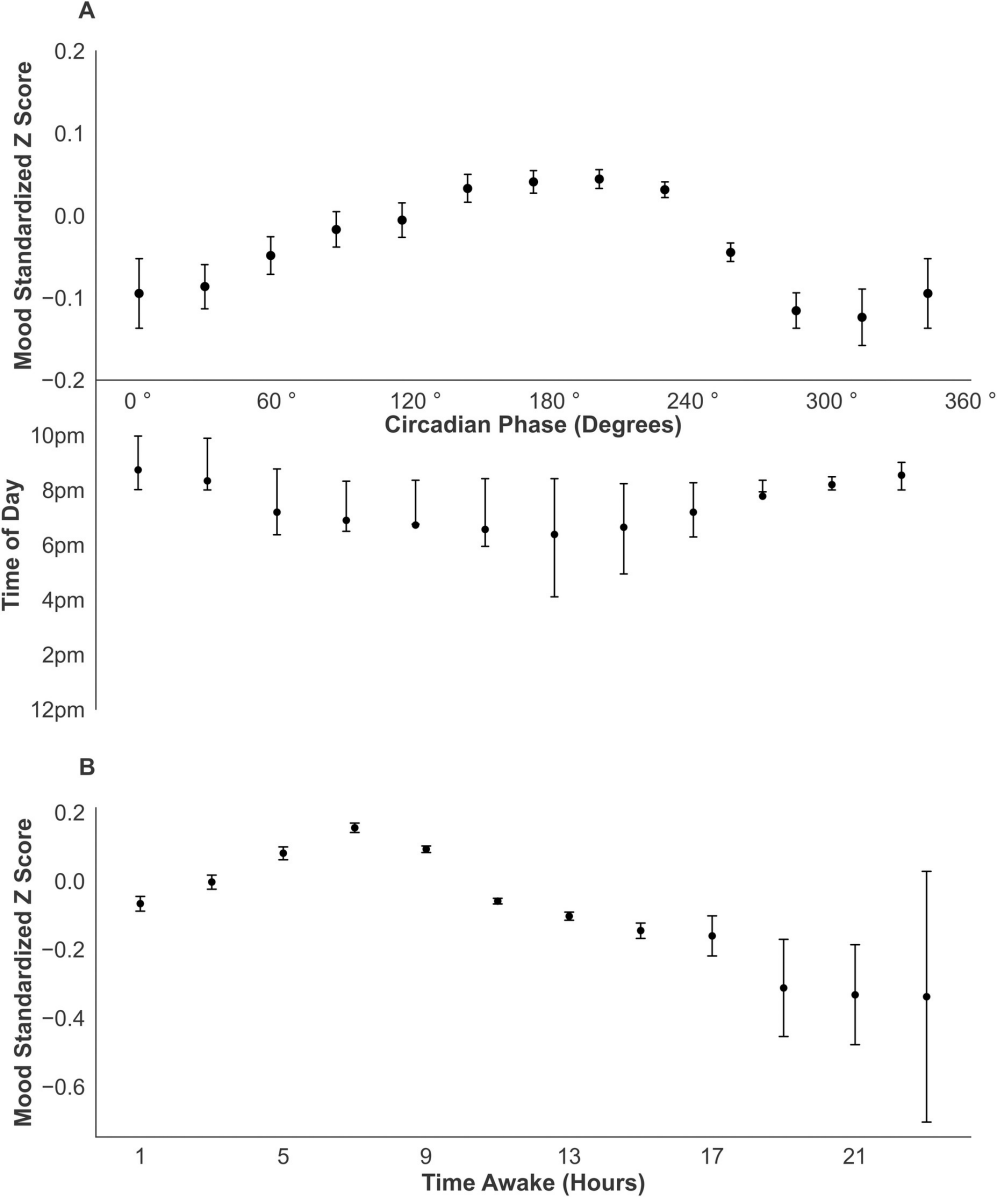
Variation in Mood with Circadian Phase and Time Awake. **(A)** Each mood score was paired with the circadian phase at time of submission. Bin edges were computed every 30° and the average standardized mood survey score was plotted against the midpoint of each bin. The median time of survey completion and interquartile range was calculated for each circadian phase bin and plotted beneath the x-axis. Bin sizes were as follows: 345°-15°: 3517, 15°-45°: 4910, 45°-75°: 6705, 75°-105°: 7463, 105°-135°: 8188, 135°-165°: 11892, 165°-195°: 18494, 195°-225°: 26778, 225°-255°: 39290, 255°-285°: 29436, 285°-315°: 8503, 315°-345°: 3135 **B)** Each mood score was paired with the corresponding number of hours the participant has been awake at survey submission. Bin edges were computed every 2 hours, and the average standardized mood survey score was plotted against the midpoint of each bin. Bin sizes were as follows: 0–2 hours: 7425, 2–4 hours: 7560, 4–6 hours: 9713, 6–8 hours:18617, 8–10 hours: 38372, 10–12 hours: 51416, 12–14 hours: 26236, 14–16 hours: 7402, 16–18 hours: 1119, 18–20 hours: 246, 20–22 hours: 180, 22–24 hours: 25.

Demographic data of age, sex, and ethnicity did not provide any additional explanatory value (p = 0.22) and were removed from the model. Average participant standardized mood scores were plotted against circadian phase in Fig 2A and against time awake in Fig 2B. Both circadian phase (p<0.001) and time awake (p<0.001) were significantly associated with mood scores (Table 2).

**Table 2:** Coefficients Fit to the Aggregate Linear Mixed Effects Model. This model tests if participant mood scores are associated with circadian phase and time awake.

Fixed Effect Coefficient	Estimate	t Value	Pr(>|t|)	Confidence Interval
Intercept (γ_00_)	0.119	15.4	<0.001	[0.103, 0.133]
Cos(phase) (γ_10_)	-0.076	-17.4	<0.001	[-0.085, -0.067]
Sin(phase) (γ_20_)	-0.024	-6.3	<0.001	[-0.032, -0.017]
Time Awake (γ_30_)	-0.016	-21.4	<0.001	[-0.018, -0.015]

The association between circadian phase and mood was rhythmic. In the fitted model, mood peaked at 198°, or approximately 5 pm on an entrained clock, and a nadir at 18°, or approximately 5 am. A significant association was also found between time awake and mood, where mood deteriorates with increasing time awake.

**Fig 3:**
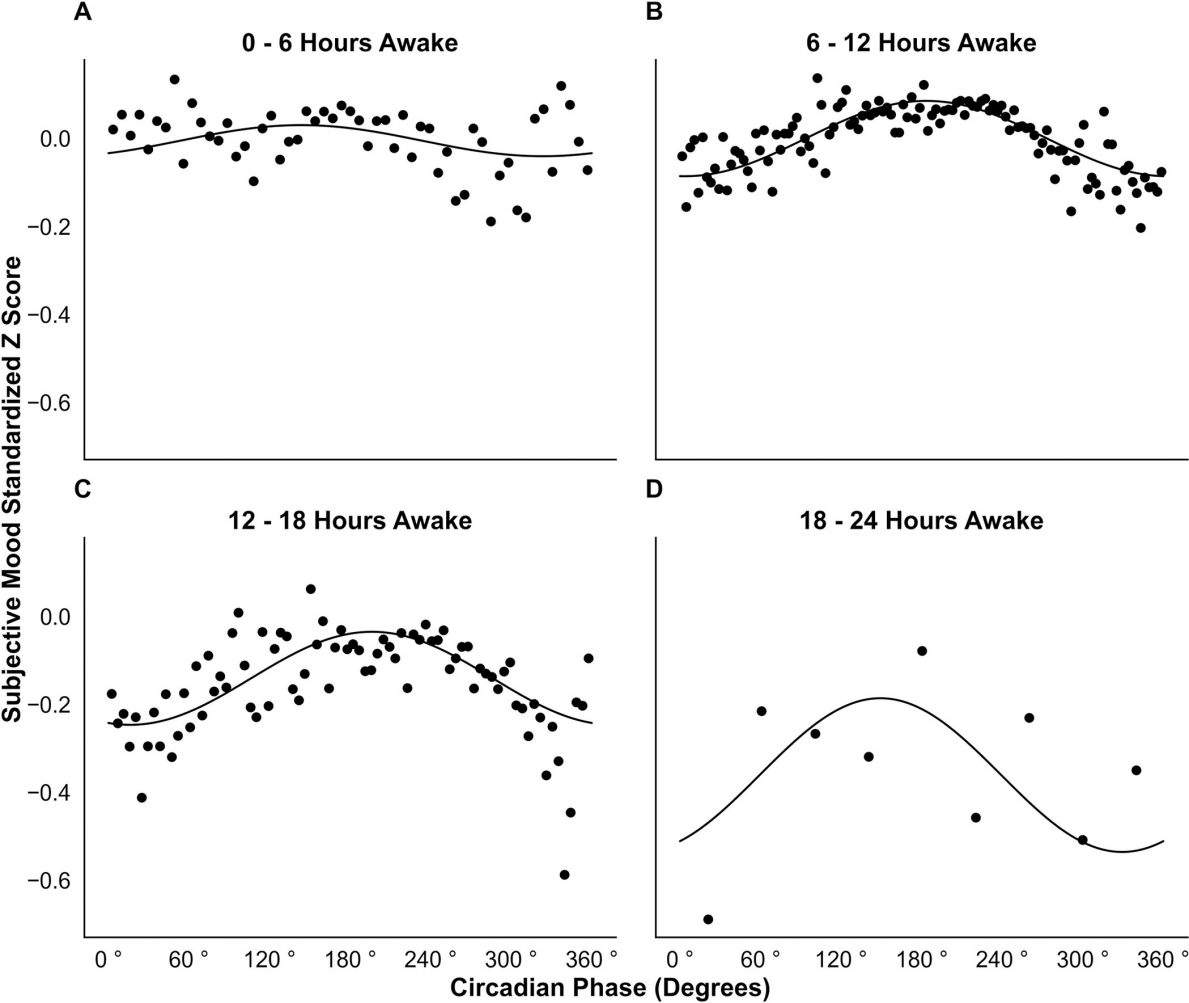
Variation in Mood with Circadian Phase Stratified by Time Awake. Survey scores were split into 4 categories of wake time: < 6 hours (**A**), 6–12 hours (**B**), 12–18 hours (**C**), and 18–24 hours (**D**). Each category was binned by circadian phase and edges computed using the Freedman Diaconis Estimator [70]. Bin resolution and average number of samples per bin for each graph are as follows: 6.5° and 449 samples (**A**), 3.0° and 919 samples (**B**), 4.5° and 434 samples (**C**), and 40° and 50 samples (**D**). The average mood z score for each bin was plotted against the bin midpoint. Linear mixed models were fit to each category. Predictions using the fixed effects of each model were plotted.

Mood scores were subsequently split into strata: 0–6, 6–12, 12–18, and 18–24 hours of time awake at survey completion. The strata had 24698, 108405, 34747, and 451 survey results respectively. Fig 3 displays each fitted model plotted with the corresponding raw data binned by circadian phase.

The median time of survey completion was calculated for each circadian phase bin. For 0–6 hours awake the median time of survey completion ranged from 1:30 pm to 8:47pm, 6–12 hours ranged from 7:23pm to 8:43pm, 12–18 hours ranged from 8:44pm to 9:43pm, and 18–24 hours ranged from 8:42pm to 9:46pm.

Circadian phase was found to be significantly associated with mood in a rhythmic form for the first three strata with *p* = 0.006, <0.0001, and <0.0001 respectively (Table 3). The fourth stratum was trending towards significance with *p* = 0.06 yet explained the most variance out of all the models (R^2^ = 0.39). The average mood standardized z scores, represented by model intercepts, were near 0 for the first two strata (-0.005 and 0.000) then progressively declined to -0.141 in the third stratum and -0.361 in the fourth stratum. Amplitude increased in a near linear fashion between models (*R*^2^ = .97) from 0.07 in the first stratum to 0.350 in the fourth stratum. In the first three strata, the nadir of mood shifted later (323°, 3°, 16° respectively) with increasing time awake.

**Table 3:** Mixed Linear Effects Model Fit to Each Strata. A linear mixed model was fit to 4 different groups of survey data: 0–6 hours, 6–12 hours, 12–18 hours, and 18–24 hours of wake time. This model tests if participant mood scores are associated with circadian phase without assuming a linear relationship with time awake.

Fixed Effect Coefficient	Estimate	t Value	Pr(>|t|)	95% Confidence Interval
0–6 Hours				
Intercept (γ_00_)	-0.005	-0.586	0.558	[-0.023, 0.012]
Cos(phase) (γ_10_)	-0.028	-2.774	0.006	[-0.049, -0.008]
Sin(phase) (γ_20_)	0.021	2.199	0.028	[0.002, 0.040]
	Amplitude	0.071	R^2^	0.0355
6–12 Hours				
Intercept (γ_00_)	0	-0.101	0.919	[-0.009, 0.008]
Cos(phase) (γ_10_)	-0.085	-15.2	<0.001	[-0.097, -0.074]
Sin(phase) (γ_20_)	-0.005	-1.07	0.285	[-0.015, 0.004]
	Amplitude	0.171	R^2^	0.008
12–18 Hours				
Intercept (γ_00_)	-0.141	16.774	<0.001	[-0.158, -0.125]
Cos(phase) (γ_10_)	-0.102	-9.909	<0.001	[-0.122, -0.081]
Sin(phase) (γ_20_)	-0.03	-3.264	0.001	[-0.047, -0.012]
	Amplitude	0.211	R^2^	0.04
18–24 Hours				
Intercept (γ_00_)	-0.361	-5.992	<0.001	[-0.158, -0.125]
Cos(phase) (γ_10_)	-0.15	-1.868	0.0625	[-0.122, -0.081]
Sin(phase) (γ_20_)	0.089	1.3	0.1942	[-0.047, -0.012]
	Amplitude	0.35	R^2^	0.39

## Discussion

Rigorous measures of circadian rhythms were previously restricted to laboratory settings. These measures included tracking core body temperature, melatonin levels, or cortisol levels [27]. Collecting this data required frequent rectal temperature measurements or consecutive blood draws. To obtain the serial measurements and avoid external environmental confounders, past studies often required participants to be confined in conditions far from what would be experienced in the natural environment. These conditions would reduce biases by controlling environmental cues such as participant caloric intake, meal timing, and ambient lighting which all can affect these measures [45, 51, 52].

Outside the laboratory, measuring temperature trends is difficult to implement as one needs to measure core body temperature throughout the day and subsequently fit an individualized oscillatory model to the collected data [53]. Using cortisol to determine circadian rhythm poses similar constraints as temperature except frequent blood draws would be needed rather than core body temperatures [54]. Dim-light melatonin onset (DLMO) has been the most promising measurement of circadian rhythm that may be extended outside the laboratory setting. In this procedure, several consecutive saliva sample can be used to determine circadian rhythm. However, this test requires the participant to sit in standardized dim lighting conditions (<20 lux) for several hours [55] which is both inconvenient and difficult to replicate in a non-laboratory setting [56].

Amplitude changes and phase shifts in temperature, cortisol, and melatonin have been utilized experimentally as markers of affective illness and treatment response [18, 57, 58]. However, the application in clinical practice has been limited due to both the inconvenience of these measurement tools and the concern that rhythmic changes can not accurately be measured in uncontrolled settings [59]. Chronotherapies have therefore been limited to broad based interventions that do not require knowledge of an individualized circadian rhythm such as total and partial sleep deprivation [60], interpersonal and social rhythms therapy for bipolar disorder which regularizes daily routines [61], and nighttime melatonin for insomnia [62].

In this study we analyzed minute by minute step, heart rate, and sleep data from 2,602 medical interns across a total of 168,311 days collected using Fitbits. From this real-world or non-laboratory data, we were able to calculate the daily circadian rhythm for each participant, information that previously would have required laboratory-based minute by minute rectal temperature monitoring or daily intravenous blood draws. Despite using these new noninvasive measures and the uncontrolled and chaotic environment of participants, we extend historic laboratory findings that mood is dependent on cumulative wake time and circadian phase in a non-linear way. We also show that measuring circadian rhythms via heart rate through wearables, or laboratory rectal temperature measurements yields similar results. More work needs to better delineate these and other circadian markers.

Our study demonstrates a clear circadian variation in mood, with peak mood occurring in the evening and troughing in the early morning. As waking hours accumulate, there is a significant decrease in mood. The circadian rhythm’s impact on mood becomes more pronounced with longer periods of wakefulness, leading to a greater rhythmic amplitude of mood.

There were several limitations to our study design. First, the mixed effects model serves as a generalized model of mood in medical interns. On an individual basis, the model only accounts for the differing variance and average mood of an individual. The inter-individual variation of mood is more complex than this and is dependent on factors such as social dynamics, schedules, and temperaments [63–65]. Second, due to the real-world nature of this data, there were minimal samples over 18 hours of time awake (N = 451), decreasing the power of the fourth model. Third, no validated emotional rating scales such as the Depression Anxiety Stress Scale or clinical screening tools were utilized in our analysis. Emotional screening tools could help identify intra participant outliers and clinical screening tools would help account for potential confounding effects of affective and anxiety disorders. Fourth, circadian phases at survey completion were not equally distributed with 66% of survey results having a circadian phase between 180° and 300° which weighted these phases higher when the model was fit. Lastly, to aid in the simplicity of our model, we left out factors such as sleep time and variability which have been recently shown to affect mood [66].

Despite these limitations, the results of our study highlight the critical role played by the circadian pacemaker in regulating mood, even during the challenging and unpredictable nature of medical internship. More so, our research shows that measuring the circadian influence on mood can be achieved noninvasively, without the need for stringent experimental conditions.

These findings have important implications in evaluating for psychopathology. It indicates clinicians should be particularly attentive to a patient’s sleep-wake cycle and the time of day of an evaluation. This raises the question of whether multiple assessments throughout various times of day may be beneficial and highlights the potential for time-of-day biases in existing literature.

The ability to measure circadian rhythm using wearable holds significant clinical promise. Psychiatric patients can be provided with a Fitbit and their circadian rhythm tracked.

Treating clinicians can monitor for circadian phase shifts as an indicator of the onset of a depressive or manic episode [67, 68] and proactively begin treatment. Furthermore, treatment response can be gauged by circadian re-alignment [24] in addition to more subjective patient reports currently utilized [69]. Chronotherapies such as social rhythms therapy and oral melatonin can be timed more accurately based on an individual’s current circadian phase.

Further work can integrate our general model of mood into more personalized mathematical models of mood. Individualized models of mood would allow clinicians to detect anomalies in patients’ emotional patterns. Clinicians could use such a model to anticipate potential mood shifts and devise intervention strategies proactively. With the combination of technology and clinical insights, such a model can pave the way for a new era in mental health care, one that is predictive, personalized, and preemptive in its approach.

## Supporting information

S1 Fig Mood survey prompt. (DOCX)
