# Supplementary figures and images for: Unraveling the interplay of circadian rhythm and sleep deprivation on mood: A Real-World Study on first-year physicians
Source: PLOS Digit Health. 2024 Jan 31;3(1):e0000439. doi: 10.1371/journal.pdig.0000439 (PMC10829990; doi:10.1371/journal.pdig.0000439)

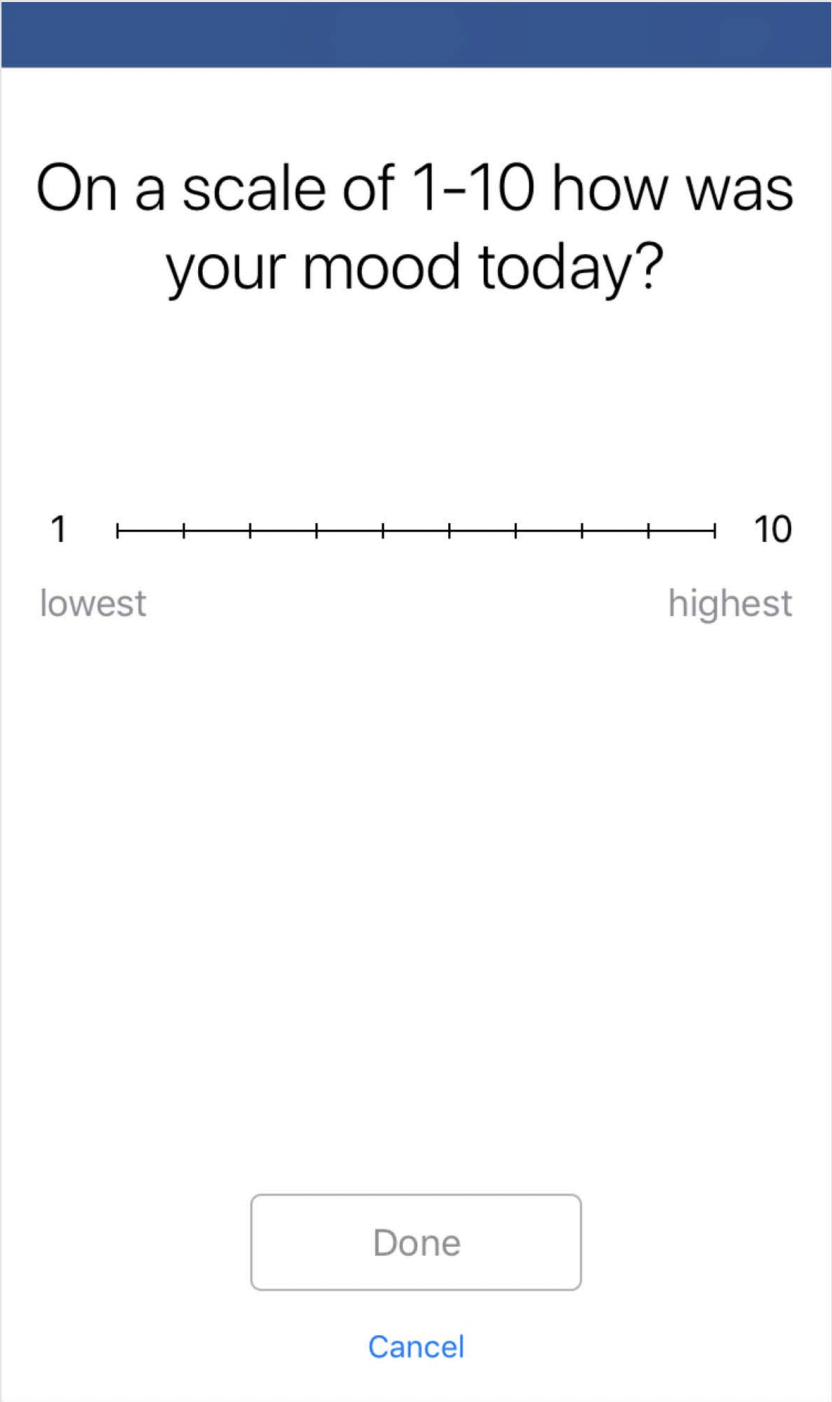


**S1 Fig:** Mood Survey Prompt

Supplement: S1 Fig — (DOCX) [file pdig.0000439.s001.docx]
